# A further analysis of olfactory cortex development

**DOI:** 10.3389/fnana.2012.00035

**Published:** 2012-08-30

**Authors:** María Pedraza, Juan A. De Carlos

**Affiliations:** Lab of Telencephalic Development (A-21), Department of Molecular, Cellular and Developmental Neuroscience, Instituto Cajal (Consejo Superior de Investigaciones Científicas)Madrid, Spain

**Keywords:** mouse, olfactory system, pallium, subpallium, Tbr1

## Abstract

The olfactory cortex (OC) is a complex yet evolutionarily well-conserved brain region, made up of heterogeneous cell populations that originate in different areas of the developing telencephalon. Indeed, these cells are among the first cortical neurons to differentiate. To date, the development of the OC has been analyzed using birthdating techniques along with molecular markers and *in vivo* or *in vitro* tracking methods. In the present study, we sought to determine the origin and adult fate of these cell populations using ultrasound-guided *in utero* injections and electroporation of different genomic plasmids into the lateral walls of the ventricles. Our results provide direct evidence that in the mouse OC, cell fate is determined by the moment and place of origin of each specific cell populations. Moreover, by combining these approaches with the analysis of specific cell markers, we show that the presence of pallial and subpallial markers in these areas is independent of cell origin.

## Introduction

The cell populations that give rise to different structures during the embryonic development of the nervous system originate in multiple and distinct germinative regions, frequently far from the site at which they ultimately settle. These cells migrate along well-established routes to occupy their final position and differentiate into adult neurons. Accordingly, neuroblast displacement is a source of cellular variability in any given structure. Neuroblasts migrate using radial and/or tangential migratory pathways. While radial movement involves the use of the radial glia as a scaffold (Rakic, 1972), tangential migration occurs independent of glial cells and follows an orthogonal route, parallel to the pial surface. This latter form of migration allows migratory cells to colonize locations at a significant distance from their origin (De Carlos et al., 1996; García-Moreno et al., 2010).

It has been proposed that each encephalic structure is comprised of multiple cell populations that are generated at diverse locations during a specific embryonic time-window, each expressing distinctive cell markers (García-Moreno et al., 2008). However, extreme caution is required when attributing a specific marker to a given cell population, which may display non-uniform expression during its spatio-temporal development. Several specific markers for distinct areas of the nervous system have been described. For example, the telencephalon is anatomically divided into the pallium and subpallium, due to the influence of dorsal (Lee and Jessell, 1999; Liem et al., 2000) and ventral (Echelard et al., 1993; Fan and Tessier-Lavigne, 1994; Martí et al., 1995) cues during development. These zones give rise to cell populations that express specific markers, such as the T-box brain 1 (Tbr1), proposed to be a typical marker of pallium-derived cells (Puelles et al., 2000). However, Tbr1 expression in pallial cells has also been described, regardless of their site of origin (Bulfone et al., 1995).

The olfactory cortex (OC) is composed of a variety of structures located in the most ventrolateral region of the mammalian telencephalon, namely the anterior olfactory nucleus, olfactory tubercle (OT), piriform cortex (PC), olfactory amygdaloid nuclei, and enthorrinal cortex (EC). Efferent projections from the olfactory bulb converge to form the lateral olfactory tract (lot), which runs through the outer portion of the PC. In rodents, the OC is one of the first structures to form in the telencephalon, even before the neocortex (García-Moreno et al., 2008). Several proliferative areas have been described from which cells migrate toward the OC, specifically colonizing the PC and OT. These include the lateral ganglionic eminence (LGE), dorsal telencephalon, rostromedial telencephalic wall, and the septoeminential sulcus. Each area gives rise to a cell population expressing specific markers in the embryo, including Tbr1, calretinin (CR), calbindin (CB), and reelin (Reln). However, the differentiation of these cells, which are derived from distinct germinative areas, and their expression during the development of the OC remain poorly understood. To better understand how this structure matures and how the distinct cell populations that contribute to this tissue are established, we have studied the expression of specific markers (Tbr1, CR, CB, and Reln) during the development of the OC.

## Materials and methods

### Animals

C57BL6 mice were raised at the animal facility of the Cajal Institute, in compliance with the current Spanish legislation (R.D. 1201/2005 and L.32/2007) and European Union Council Guidelines (2003/65/CE) concerning the care and use of experimental animals. The day of detection of the vaginal plug was considered embryonic day 0 (E0). Embryos were anesthetized by hypothermia, while pregnant mice (*n* = 10) were anesthetized by intraperitoneal injection with Equithesin (3 mL/kg body weight). Thirty embryos were used for immunohistochemistry studies and an additional 20 were electroporated *in utero*. The distribution of the animals used and their survival time is shown in the table below.

**Table:** 

	Pregnant mice	E11	E12	E14	E18	P15	
Immunohistochemistry	7		8	8	8	6	
*In utero* experiments	3	20	Survival until P15				75%

### Ultrasound-guided *in utero* plasmid injections and electroporation

The plasmids used were pPB-Ubc-EGFP and mPBase (transposase), kindly provided by Prof. A. Bradley [Cambridge, UK; plasmid described in Yusa et al. (2009)]. The GFP-plasmid contains specific regions recognized by the transposase that facilitates its integration into the genome. Using an ultrasound guided injection system (VeVo 770®:VisualSonics Inc. Toronto, Canada), embryos were injected to specifically label newly generated cells (pallium or subpallium). Briefly, E10–E12 pregnant mice were anesthetized with isoflurane (Isova vet, ref. 240055: Centauro, Barcelona, Spain), their uterine horns were exposed through the abdominal wall and they were covered with pre-warmed ultrasound gel (Parker Laboratories Inc., NJ, USA). A volume of 1–2 μl of the recombinant plasmid solution was injected in the lateral ventricle of each embryo and they were electroporated with 5 pulses (50 ms) using a BTX Electroporator ECM 830 (BTX: MA, USA). Electroporation was achieved by discharging a 500 μF capacitor charged to 25 V with a sequencing power supply via a pair of round platinum plates (5 mm diameter). The uterine horns were then set back into the abdominal cavity, which was filled with warm physiological saline, and the abdominal muscle and skin were closed with silk sutures. After surgery, pregnant mice received a subcutaneous injection of the antibiotic enrofloxacine (Baytril, 5 mg/Kg: Bayer, Leverkusen, Germany) and an intraperitoneal injection of the anti-inflammatory/analgesic ketorolac (Droal, 300 μg/Kg VITA Laboratories, Barcelona, Spain). The injected embryos were transcardially perfused at postnatal stages, with 4% paraformaldehyde (PF) in 0.1 M phosphate buffer (PB, pH 7.2), and their brains were removed, embedded in agar, and coronal sectioned at 50 μm with a vibratome.

### Immunohistochemistry

Single and dual immunohistochemistry was performed as described previously (García-Moreno et al., 2008), using the following primary antibodies: mouse-anti-Reelin (1:1000; MAB5364, Clone G10, Chemicon; Temecula, CA); rabbit-anti-Calbindin-D28K (1:10,000; CB38, Swant, Bellinzona, Switzerland); rabbit-anti-Calretinin antiserum (1:2000; 7699/4, CR, Swant); rabbit-anti-Tbr1 (1:1000; AB9616, Chemicon); rat-anti-GFP (1:20,000; 4404-84, Nacalai Tesque, Kyoto, Japan).

The following secondary antibodies were used: Alexa 568 goat-anti-rabbit IgG (1:2000; A11011, Molecular Probes); Alexa 568 anti-mouse IgG (1:2000; A11004, Molecular Probes); Alexa 488 anti-rat (1:2000; A11034, Molecular Probes).

For all antibodies a series of control sections was processed without the primary antibody in which no specific staining was detected. Sections were mounted on gelatinized slides and counterstained with 0.002% bisbenzimide in PBS (Hoechst 33258: Sigma, St. Louis, MO).

### Equipment and settings

Injected embryos were examined under a fluorescence-dissecting microscope (Leica MZFL-III) and after mounting with a mixture of glycerol-phosphate buffer (PB, 1:1), fluorescent sections were analyzed under a fluorescent microscope (Nikon, Eclipse E600) equipped with a digital camera (Nikon DMX 1200F) and the appropriate filter cubes: rhodamine (569–610 nm) and fluorescein (450–490 nm), to visualize Alexa 568 and GFP/Alexa 488, respectively. Bisbenzimide labeling was analyzed with ultraviolet illumination.

## Results

### Temporal expression patterns in the olfactory cortex

To analyze the spatio-temporal expression of different proteins during the development of the OC, we performed immunohistochemistry for the following markers at E12, E14, E18, and in adulthood: Tbr1, CR, CB, and Reln.

#### Tbr1 expression during olfactory cortex development

**Figure 1:**
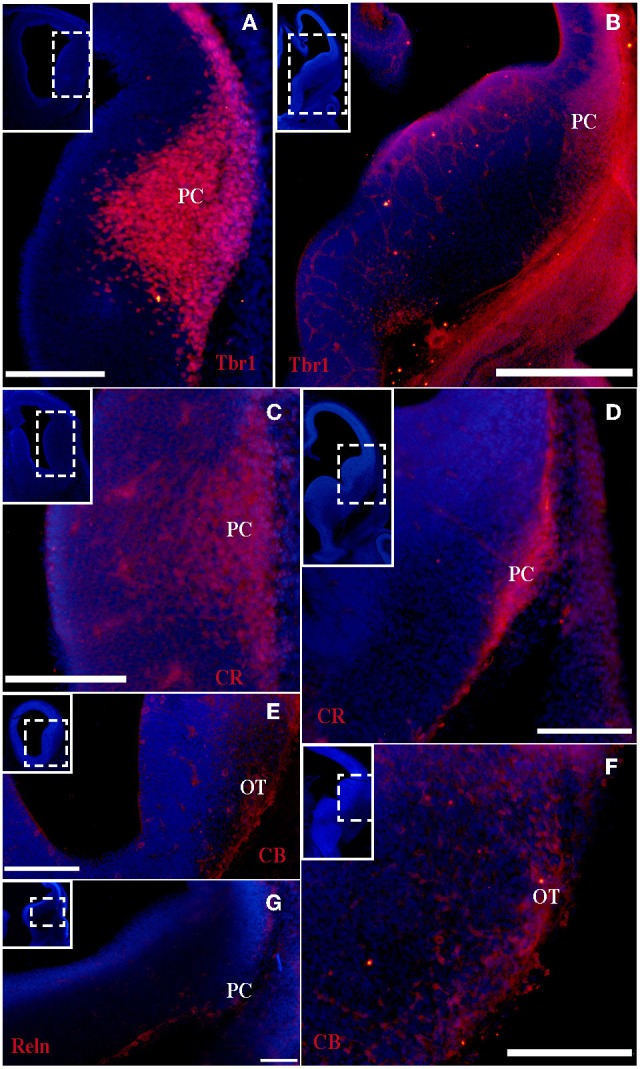
**Tbr1, CR, CB, and Reln (red) expression in the olfactory cortex at E12. (A–D)** Tbr1 and CR expression in the rostral and caudal regions of the piriform cortex. **(E,F)** CB is expressed in the OT but not the PC. **(G)** Reln expression is restricted to the outermost portion of the PC. Coronal sections; midline is to the left and dorsal is up. Blue corresponds to the bisbenzimide counterstaining. Scale bars: **A–F**, 500 μm; **G**, 100 μm.

**Figure 2:**
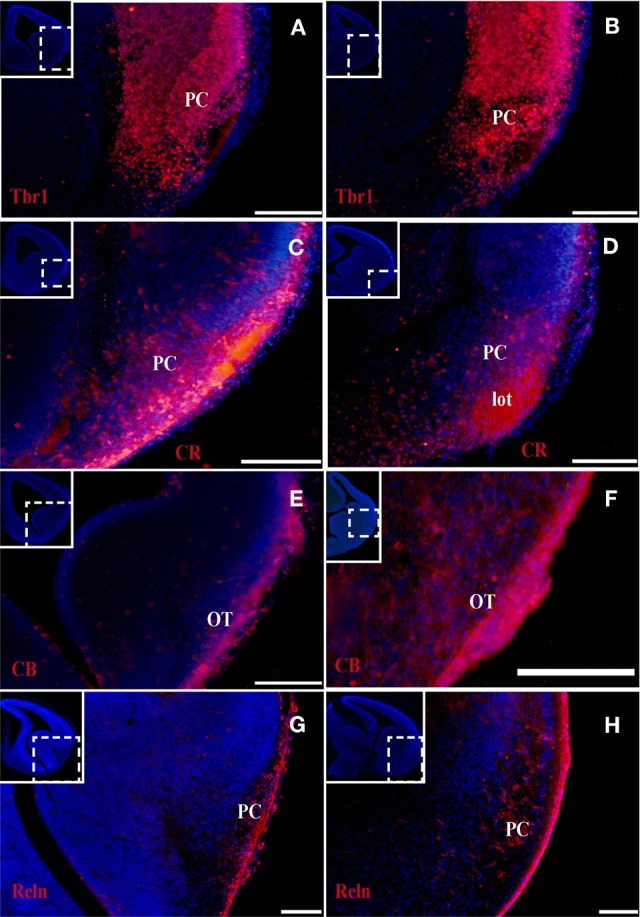
**Tbr1, CR, CB, and Reln (red) expression in the olfactory cortex at E14. (A–D)** Tbr1 and CR expression in the rostral and the caudal regions of the piriform cortex. CR expression in the lot **(D)**. **(E,F)** CB is expressed in the OT but not the PC. **(G,H)** Reln expression is restricted to the outermost portion of the PC. Coronal sections; midline is to the left and dorsal is up. Blue corresponds to the bisbenzimide counterstaining. Scale bars: **A–F**, 500 μm; **G** and **H**, 100 μm.

**Figure 3:**
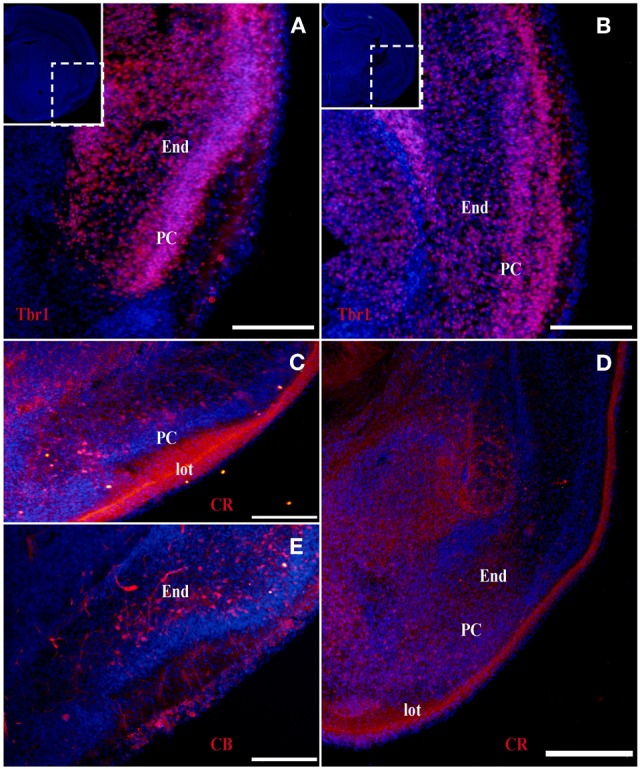
**Tbr1, CR, and CB (red) expression in the olfactory cortex at E18. (A–D)** Tbr1 and CR expression in the rostral and the caudal regions of the piriform cortex. Both Tbr1 and CR are expressed in the End **(A,B,D)**, whereas only CR is expressed in the lot **(C)**. **(E)** CB is only expressed in the End. Coronal sections; midline is to the left and dorsal is up. Blue corresponds to the bisbenzimide counterstaining. Scale bars: 500 μm.

**Figure 4:**
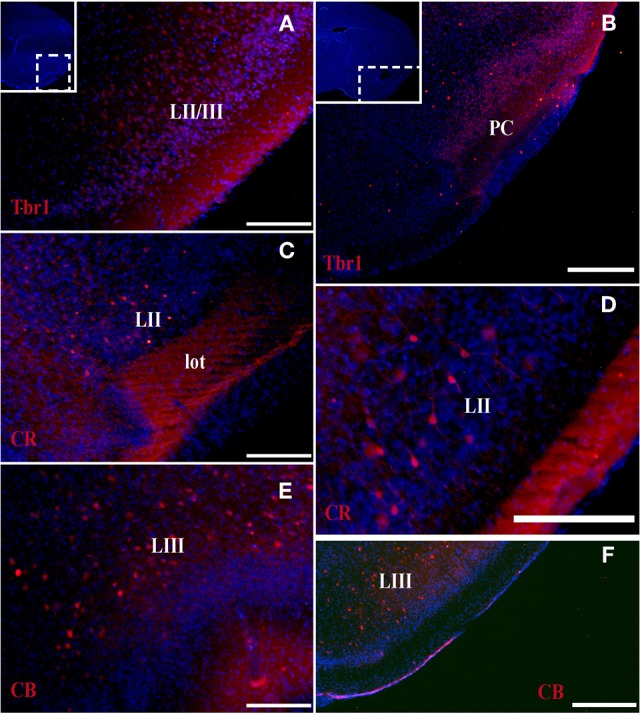
**Tbr1, CR, and CB (red) expression in the olfactory cortex in adult mice. (A,B)** Tbr1 expression in layers II and III of the PC. **(C,D)** CR is expressed in layer II of the PC and in the lot. **(E,F)** CB expression is restricted to the layer III in the piriform cortex. Coronal sections; midline is to the left and dorsal is up. Blue corresponds to the bisbenzimide counterstaining. Scale bars: 500 μm.

At the earliest selected embryonic stage, E12, significant Tbr1 expression was observed in the rostral and caudal areas of the PC (Figures 1A,B), and in the mantle zone of the LGE, but not in the OT. At E14, Tbr1 expression was clearly detected in both the cortical neuroepithelium and the PC (Figures 2A,B), and Tbr1 was consistently expressed from the rostral to caudal portions of the PC. At E18, Tbr1 expression was restricted to the PC and endopiriform nucleus (End; Figures 3A,B), and finally, Tbr1 expression was restricted to layers II and III in the mature PC (Figures 4A,B).

#### CR expression during olfactory cortex development

The expression pattern of CR observed at E12 was similar to that of Tbr1, albeit more restricted to the PC (Figures 1C,D), with no labeling observed in the OT. CR expression persisted in the PC at E14 (Figures 2C,D) and this protein was also detected in the lot (Figure 2D). CR expression in the lot was maintained at E18 (Figures 3C,D) and in adult mice (Figure 4C), as well as in layer II of the PC (Figures 4C,D).

#### CB expression during olfactory cortex development

In contrast to Tbr1 and CR, at E12 (Figures 1E,F) and E14 (Figures 2E,F) CB expression was restricted to the OT, with no labeling detected in the PC. Similarly, unlike Tbr1 and CR, CB expression was limited to the End at E18 (Figure 3E) and in adulthood, CB expression was limited to layer III of the PC (Figures 4E,F).

#### Reln expression during olfactory cortex development

Reln expression at E12 was limited to the most superficial portion of PC (Figure 1G). At E14, this pattern had changed and Reln occupied the inner portion of the PC (Figures 2G,H). A global increase in Reln expression was observed at later stages.

### Markers associated with sites of cell origin

Because cell tracers did not allow all pallial/subpallial-derived cells to be identified, we tracked cells originating in the pallial or subpallial areas and characterized them at the molecular level upon reaching their destiny.

In order to mark the injection site (site of cell origin) throughout our lengthy experiments, we electroporated a GFP-expressing plasmid that becomes integrated into the genome of the cells. If integration is not achieved, the tagged plasmid is diluted over successive cell divisions and ultimately disappears, providing a means of identifying the precise area where electroporation occurred (site of origin of the labeled cells).

**Figure 5:**
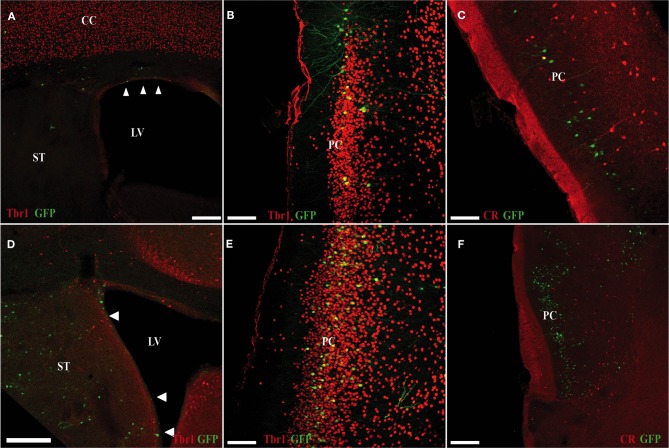
**Electroporation of a GFP plasmid into pallial (A–C) and subpallial (D–F) areas of E11 mice (sacrificed at P5). (A–C)** Many of GFP cells that originate in the ventricular zone of the pallium (green) do not co-express Tbr1 (red; **A,B**). **(C)** Pallial-derived GFP cells do not express CR (red). **(D–F)** Many of the GFP-cells that originate in the ventricular zone of the subpallium (green) co-express Tbr1 (red; **D,E**). **(F)** Subpallial-derived cells do not express CR (red). Coronal sections; midline is to the right and dorsal is up. Scale bars: **A,D**, 200 μm; **B,C,E,F**, 100 μm.

At P15, GFP cells transfected in the pallium at E11 were detected in the OC (Figures 5A–C). Despite being generated in pallium (with no contamination from subpallial areas; Figure 5A), we observed few GFP cells co-expressing Tbr1, which has been proposed as a marker of pallial-generated cells (Figures 5A,B), and we detected no pallial-generated cells expressing CR (Figure 5C). Unexpectedly, after electroporation of cells generated in the subpallium (Figure 5D), most GFP cells co-expressed Tbr1 (Figure 5E) but not CR (Figure 5F) in the OC.

## Discussion

### Restriction and specificity of markers in different areas during the development of the olfactory cortex

Marker expression in distinct telencephalic cell populations varies during embryonic development and adulthood. This reflects either the cellular contribution of different germinative areas, the rearrangement of distinct cell populations, and the silencing or new expression of specific markers.

The formation of the OC begins early in development and continues throughout the embryonic stages. This region is comprised of several structures that are formed by many cell populations that originate in diverse areas of the telencephalon (García-Moreno et al., 2008), resulting in significant phenotypic variability. Accordingly, the different markers analyzed are not expressed constantly throughout development. We previously demonstrated that at early developmental stages, Tbr1, Reln, and CR serve as specific markers of the presumptive PC, while CB is expressed in the presumptive OT (García-Moreno et al., 2008). In the present study, we performed a temporal analysis of these markers from early murine developmental stages through adulthood.

Tbr1 expression was relatively constant throughout development, and was initially expressed in the presumptive PC before it was subsequently limited to layers II and III in adulthood. CR expression was constant in the PC throughout development, but was restricted to layer II and the lot in the adult OC. By contrast, CB labeling was absent in the embryonic OC, but was expressed in layer III of the PC in adult mice. Reln expression was profusely observed in early embryonic stages and becomes diffuse in late stages.

In summary, because Tbr1 and CR expression remain constant throughout the development of the OC, they represent valid and useful markers of specific structures within the OC. The same cannot be said of CB and Reln, due to their fluctuating expression during the development of the OC.

### Tbr1 as a useful structural marker but is not a reliable marker of pallial-derived cells

The origins of diverse brain structures remains a contentious issue, in part because most studies are based on the use of specific markers, the expression of which may fluctuate during development. We propose that these currently used tracking methods are inadequate and as such, we have tracked the development of two specific cell populations derived from the pallium and subpallium. Although electroporation labels a larger area than that labeled using tracers (such as CFDA) or retroviruses, this experimental approach allows the origin of the labeled cells to be identified (taking into account that we labeled either pallial or subpallial origins, but not both), which is not possible using the other methods cited above. Thus, this technique allowed us to analyze the migration of some cell populations from their site of generation to the OC.

While Tbr1 has been proposed as a specific marker of pallium-derived cells (Puelles et al., 2000), our findings demonstrate that the OC contains many cells of pallial origin that do not express this marker. Moreover, we found that many cells originating in subpallium express this marker in the OC. Taken together, these results underscore the importance of using cellular tracking methods to determine the origin of different cell populations, instead of relying solely on the expression patterns of specific cell markers.

### Conflict of interest statement

The authors declare that the research was conducted in the absence of any commercial or financial relationships that could be construed as a potential conflict of interest.
